# Thoracic aortopathy in Turner syndrome and the influence of bicuspid aortic valves and blood pressure: a CMR study

**DOI:** 10.1186/1532-429X-12-12

**Published:** 2010-03-11

**Authors:** Britta E Hjerrild, Kristian H Mortensen, Keld E Sørensen, Erik M Pedersen, Niels H Andersen, Erik Lundorf, Klavs W Hansen, Arne Hørlyck, Alfred Hager, Jens S Christiansen, Claus H Gravholt

**Affiliations:** 1 Medical Department M (Endocrinology and Diabetes) and Medical Research Laboratories, Aarhus Sygehus NBG, Aarhus University Hospital, DK-8000 Aarhus C, Denmark; 2 Department of Cardiology, Skejby Sygehus, Aarhus University Hospital, DK-8200 Aarhus N, Denmark; 3 The MR Centre, Skejby Sygehus, Aarhus University Hospital, DK-8200 Aarhus N, Denmark; 4 Department of Medicine, Silkeborg Centralsygehus, DK-8600 Silkeborg, Denmark; 5 Department of Radiology, Skejby Sygehus, Århus University Hospital, DK-8200 Aarhus N, Denmark; 6 Department of Pediatric Cardiology and Congenital Heart Disease, Deutsches Herzzentrum München, Technische Universität München, D-80636 München, Germany

## Abstract

**Background:**

To investigate aortic dimensions in women with Turner syndrome (TS) in relation to aortic valve morphology, blood pressure, karyotype, and clinical characteristics.

**Methods and results:**

A cross sectional study of 102 women with TS (mean age 37.7; 18-62 years) examined by cardiovascular magnetic resonance (CMR- successful in 95), echocardiography, and 24-hour ambulatory blood pressure. Aortic diameters were measured by CMR at 8 positions along the thoracic aorta. Twenty-four healthy females were recruited as controls. In TS, aortic dilatation was present at one or more positions in 22 (23%). Aortic diameter in women with TS and bicuspid aortic valve was significantly larger than in TS with tricuspid valves in both the ascending (32.4 ± 6.7 vs. 26.0 ± 4.4 mm; p < 0.001) and descending (21.4 ± 3.5 vs. 18.8 ± 2.4 mm; p < 0.001) aorta. Aortic diameter correlated to age (R = 0.2 - 0.5; p < 0.01), blood pressure (R = 0.4; p < 0.05), a history of coarctation (R = 0.3; p = 0.01) and bicuspid aortic valve (R = 0.2-0.5; p < 0.05). Body surface area only correlated with descending aortic diameter (R = 0.23; p = 0.024).

**Conclusions:**

Aortic dilatation was present in 23% of adult TS women, where aortic valve morphology, age and blood pressure were major determinants of the aortic diameter.

## Background

Women with Turner syndrome (TS) face a significant risk of premature cardiovascular death due to aortic dissection, ischemic heart disease and stroke [1]. The high prevalence of congenital cardiovascular malformations [2,3] and hypertension also contributes adversely to the increased cardiovascular morbidity and mortality [4]. Careful and continuous monitoring of the aorta in this patient group is therefore of vital importance [5] and cardiovascular magnetic resonance (CMR) may provide superior imaging compared with echocardiography [6]. Aortic dilation has been found in 12-32% [6-9] of relatively young women with TS. Since the final height of women with TS is reduced, it may well be needed to correct for this potential confounder when analysing aortic measurements and adjusting for body surface area (BSA) has been performed on data from women with TS [8]. Indexing to segments of the aorta that are unaffected by disease and reflect normal aortic size for the individual has also been used ("aortic diameter index") [8,9]. Recent echocardiographic studies in women with TS have strongly indicated that dilation of the aortic root particularly occurs in those with bicuspid aortic valve (BAV) [10,11]. However, the combined impact of BAV, and other risk factors such as blood pressure, repaired coarctation, body composition and dysmetabolism on the thoracic aorta in women with TS has to our knowledge not been examined.

We therefore studied to what extent aortic dilatation, the presumed forerunner of dissection, in TS is influenced by such risk factors. Accordingly, the aims of the present study performed in a group of women with TS were to investigate aortic dimension in relation to aortic valve morphology, BSA and other measures of anthropometry, blood pressure and clinical characteristics typical for TS and to establish normative data on aortic diameter in TS.

## Methods

One hundred and two women (aged 38 ± 11 years, range: 18-62 years) with TS verified by karyotyping (45,X: n = 58 (57%); other karyotypes (mosaics, isochromosomes): n = 44 (43%)) were included consecutively through the National Society of Turner Contact Groups in Denmark and the local Out-patient clinic. Patients with malignant disease, clinically significant liver disease and mechanical heart valves were excluded. Twenty-four healthy women (aged 43 ± 10 years, range: 25-63) taking no daily medication (oral contraceptives accepted), recruited through advertising, served as controls. The protocol was approved by the Aarhus County Ethical Scientific Committee (# 20010248) and complies with the Declaration of Helsinki. Informed consent was obtained from all participants.

All trial participants were admitted to the research laboratory at 8 am after an overnight fast (10 h). Body weight was measured to the nearest 0.1 kilogram (kg) and body height was measured to the nearest 0.5 cm. The presence of webbed neck and/or low hairline was noted. Previously diagnosed hypertension was registered. Information on the age at menarche, age when exogenous estrogen was introduced, age at premature menopause, and duration of hormone replacement therapy (HRT) were registered, enabling summation of total estrogen exposure (in years). The duration of estrogen insufficiency were estimated as the number of years between the age of 13 years and 53 years, during which participants neither were taking HRT, nor had spontaneous menstrual bleedings. Due to claustrophobia (n = 5), cochlear implant (n = 1) and technical problems (n = 1) CMR was performed on 95 of 102 participants. Echocardiography was performed in 101 of 102. Echocardiography and CMR were completed for all control women.

### Cardiovascular Magnetic Resonance

All examinations were performed on a 1.5 Tesla whole body MR scanner (ACS-NT, Philips Medical Systems; maximum gradient performance 30 mT/m amplitude, slew rate 150 T/m/s) using a commercially available 5-element cardiac coil. After initial scouts a 3D block of data (27 cm (AP) × 15 cm (FH) × 36 cm (LR)) covering the ascending aorta, aortic arch and descending thoracic aorta was acquired. A nearly isotropic 3D steady-state free precession (SSFP) navigator and ECG triggered sequence acquired in diastole during free breathing was used [12]. The voxel size was 1.4 mm × 1.4 mm × 1.5 mm. Since only extra-cardiac vessels were studied the length of the diastolic acquisition window was increased to approximately 250 ms. This allowed the total scan time of 8-12 min, depending on the heart rate and breathing pattern. In the attempt to minimize effective time in the scanner to the trial subject, the acquisition window was set at 230 ms. This resulted in capturing of end-diastole in some patients, with a resulting inferior image quality in a fraction of the patient at the most dynamic portion of the aorta (sinus and hinge level). As a result, we refrained from measurement of these positions.

**Figure 1:**
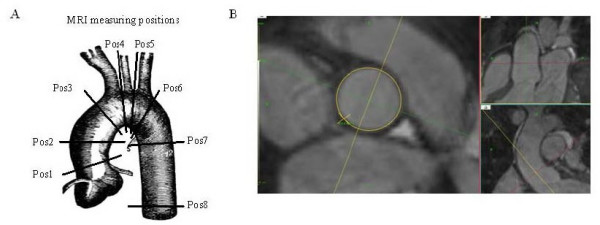
**CMR measuring positions, where pos1, pos3 and pos 6 were also measured by echocardiography**. Post-processing of 3D isotropic CMR in positon 1 of the ascending aorta, at sinotubular level. It illustrates the ability to ensure precise and reproducible measurement of aortic diameter, where accuracy is ensured by the use of 3 separate planes placed perpendicular to each other and the aortic wall at the position of measuremtn.

All aortic measurements were obtained with dedicated prototype software (Systematic Software Engineering, Aarhus, Denmark) that allowed real time reconstruction of any plane in the 3D stack of data. Any chosen plane was then interactively positioned across the aorta at the following 8 positions (pos): Sinotubular junction (pos1); ascending aorta midway between the sinotubular junction and the innominate artery (pos2); ascending aorta immediately proximal to innominate artery (pos3); proximal transverse arch midway between innominate and left carotid artery (pos4); distal transverse arch just proximal to left subclavian artery (pos5); aortic isthmus immediately distal to the left subclavian artery (pos6); descending aorta between left pulmonary artery and top of left atrium (pos7) and descending aorta at the most caudal border of the left atrium (pos8) (Figure 1A).

To visualize anatomical landmarks and secure correct angulation relative to the aortic wall, especially in the aortic arch, two imaging planes orthogonal to each other as well as the plane of measurement were displayed simultaneously while defining the plane of measurement during the navigation in the 3D data block (Figure 1B). For locations defined by the branching of an artery, the position was defined as the plane rectangular to the aorta as close to the branching artery as possible without any sign of branching visible. The largest diameter was measured manually at each plane of measurement. The largest diameter was used in subsequent comparisons [12].

Cut-off levels for dilation and aneurysm were calculated from the CMR diameters in the control population. Dilation was defined as a diameter exceeding the mean + 2SD with aneurysm defined as a diameter larger than 1.5 times the mean. The ratio of the ascending and descending aortic dimensions relative to position 8 was calculated and a ratio greater than 1.5 defined as aortic dilation. Both methods of defining aortic dilation were used in the data evaluation.

### Echocardiography

Echocardiographic examinations were performed by the same observer on a GE Vivid Five (GE Medical System, Horten, Norway) with a 2.5 MHz transducer and using second harmonic modalities [13]. Aortic diameters were measured at pos1, pos3 and pos6 (Figure 1A) and dilation at the sinotubular junction (pos 1) defined as a diameter above 32 mm [14]. Finally, aortic valve morphology was determined and characterized as tricuspid (TAV), BAV or undetermined.

### Ambulatory blood pressure

Twenty-four hour ambulatory blood pressure (AMBP) was recorded with a Spacelabs 90207 (Redmond, Washington, USA), using oscillometric technique. Blood pressures were measured from the left upper arm, using an individualised cuff size, and readings were obtained every 20 minutes, with day- and night-time defined from diary-registered bed and rise-time. Hypertension was defined as a mean day time AMBP > 130/85 mmHg and/or night-time values > 110/70 mmHg, which in an outcome driven study corresponds to the cardiovascular risk of a clinic blood pressure greater than 130/85 mmHg [15]. Hypertension was defined independently of antihypertensive treatment.

### Statistical methods

Statistical calculations were done using SPSS 15.0. Normality of data, including Levene's test for equality of variances, was tested with the Shapiro-Wilk test of normal distribution. Correlation analysis between aortic dimensions and normally distributed parameters was performed with Pearson's coefficient of correlation, while Spearman's rank correlation analysis was used to examine the relation between non-normally distributed data. The χ^2 ^test was used to test difference between groups, using the Fisher's exact test if group number were < 5. Multiple backward stepwise linear regression models were constructed to examine the principal determinants of aortic dimensions, where independent variables were omitted from the model when p > 0.1. P-values less than 5% were considered significant.

Aortic diameters adjusted for BSA or height as suggested by Ostberg et al [8], did not influence the results significantly. Diameters adjusted for BSA are reported.

## Results

Characteristics of participants are listed in table 1. The control subjects were taller, heavier, and had higher BSA, but similar BMI. Fasting glucose, HbA1C, and lipids were comparable between groups (Table 1). In the TS group 57 (58%) were of 45,X karyotype and 43 (44%) were of other karyotypes. Among the women with TS the median duration of HRT was 19 years (ranging from none to 36 years), and the median time of HRT deficiency was 3 years (ranging from none to 40). Twelve of 81 patients in the age-group 18-53 had chosen never to receive HRT, seven of these had spontaneous menstruation.

**Table 1:** Clinical characteristics of participants.

	TS				Controls			
	
		N	Mean ± SD	Range	n	Mean ± SD	Range	p-value
Age (years)		102	37.7 ± 10.9	18 - 62	24	42.7 ± 10.4	25 - 63	0.04
Height (cm)		102	147.3 ± 7.1	134 - 171	24	168.2 ± 5.9	155 - 177	< 0.001
Weight (kg)		101	57.6 ± 12.4	34 - 104	24	69.4 ± 7.3	57 - 84	< 0.001
BMI (kg/m^2^)		101	26.6 ± 5.6	16 - 48	24	24.6 ± 3.2	21 - 34	0.1
BSA (m^2^)		101	1.49 ± 0.16	1.2 - 2	24	1.79 ± 0.092	1.6 - 2	< 0.001
24 h AMBP (mm Hg)	Systolic	97	122 ± 14	99 - 192	24	115 ± 9	100 - 133	0.03
	Diastolic	97	77 ± 11	54 - 134	24	73 ± 7	63 - 87	0.1
Estrogen deficiency (yrs)		94	6.4 ± 8.3	0 - 40				
GH treatment (years)		23	5.5	1 - 12				
Antihypertensive treatmeant (years)		24	5.3	0.5 - 19				

Baseline data in TS and healthy female control subjects.

AMBP are average values of 24-hour ambulatory blood pressure registration (day and night time values not shown).

### Aortic valve morphology

BAV was found in 26 of 102 (25%) women with TS, and a tricuspid aortic valve was seen in 73 women. Aortic valve morphology could not be accurately defined in 3 (3%). None of the controls had tricuspid aortic valves.

### Blood pressure and aortic coarctation

Hypertension was present in 55/96 (58%) of women with TS and in 9/24 (38%) of the controls. In women with TS, hypertension was present in 39/73 (53%) of those with a tricuspid valve and in 15/26 (58%) with BAV. Repaired aortic coarctation was more frequent in TS women with BAV (5/24, 21%) than it was with a tricuspid aortic valve (3/65, 5%).

### Aortic diameters

The absolute aortic diameters obtained by CMR in the TS population was not different in the ascending aorta compared to the control group (Table 2). However, a wider range and greater standard deviation was seen in TS. At the distal transverse aortic arch (pos5) and aortic isthmus (pos6) the diameter was smaller in TS (Table 2).

**Table 2:** Aortic dimensions obtained with CMR in TS and controls. TS dimensions adjusted for BSA are shown.

	CMR TS		CMR controls		TS vs C	CMR TS_korrBSA_	TS_korrBSA _vs C
	
	n	Mean ± SD (range)(mm)	n	Mean ± SD (range)(mm)	P-value	Mean ± SD (range)(mm)	P-value
Sinotubular junction	95	25.4 ± 4.7 (17-39)	24	26.4 ± 2.3 (22-32)	0.4	26.2 ± 4.6 0.8	0.8
Ascending aorta	95	27.4 ± 5.7 (16-48)	24	27.6 ± 2.7 (22-32)	0.9	28.2 ± 5.7 0.6	0.6
Aorta proximal to innominate artery	95	25.4 ± 4.0 (18-38)	24	26.3 ± 2.4 (22-31)	0.3	26.3 ± 4.0 1.0	1.0
Prox. transverse aortic arch	73	23.5 ± 3.7 (17-34)	20	24.9 ± 2.2 (21-30)	0.1	24.5 ± 3.7 0.6	0.6
Distal transverse aortic arch	94	20.5 ± 2.6 (14-29)	22	23.5 ± 1.9 (20-27)	< 0.001	21.6 ± 2.6 0.001	0.001
Aortic isthmus	94	19.3 ± 2.4 (14-28)	23	22.2 ± 2.0 (18-26)	< 0.001	20.2 ± 2.4 < 0.001	< 0.001
Descending aorta	95	19.4 ± 2.9 (14-31)	24	20.2 ± 1.8 (17-26)	0.2	21.1 ± 2.8 0.1	0.1
Descending aorta (diaphragm level)	94	18.2 ± 2.4 (13-27)	24	18.9 ± 1.9 (16-24)	0.2	20.1 ± 2.4 0.02	0.02

Women with TS and BAV had significantly larger diameters at all positions except from pos5 and pos6, when compared to those with a tricuspid valve (Table 3). A subgroup analysis of aortic diameter in women with TS and no coarctation (n = 79) revealed a significantly larger diameter of the ascending aorta (pos1, pos2 and pos4) but not the descending aorta (pos7, pos8) in the BAV subgroup compared with the TAV subgroup.

**Table 3:** Aortic dimensions in TS with bicuspid (TS_bicuspid_) and tricuspid(TS_tricuspid_) aortic valves.

	CMR TS_bicuspid_		CMR TS_tricuspid_		TS_tricuspid _vs TS_bicuspid_
	
	n	Mean ± SD (range)(mm)	n	Mean ± SD (range)(mm)	P
Sinotubular junction	23	29.1 ± 5.4 (20-39)	66	24.4 ± 3.8 (17-36)	< 0.001
Ascending aorta	23	32.4 ± 6.7 (21-48)	66	26.0 ± 4.4 (16-38)	< 0.001
Aorta proximal to innominate artery	23	27.6 ± 4.6 (21-38)	66	24.7 ± 3.6 (18-34)	0.002
Prox. transverse aortic arch	19	25.8 ± 4.8 (18-34)	50	22.8 ± 3.0 (17-30)	0.002
Distal transverse aortic arch	23	20.6 ± 3.5 (14-29)	65	20.4 ± 2.3 (15-27)	0.8
Aortic isthmus	23	19.7 ± 3.2 (15-28)	65	19.1 ± 2.1 (14-24)	0.3
Descending aorta	23	21.4 ± 3.5 (17-31)	66	18.8 ± 2.4 (14-24)	< 0.001
Descending aorta (diaphragm level)	23	19.7 ± 2.8 (16-27)	65	17.7 ± 2.1 (13-23)	0.001

### Aortic ratio

**Figure 2:**
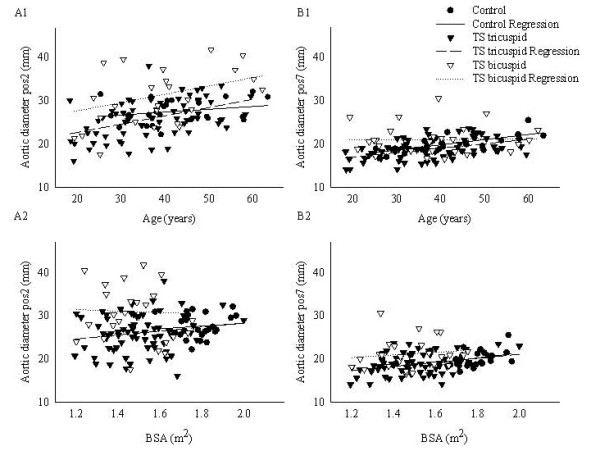
**Correlations between ascending (pos2) (A, left panel) and descending (pos7) (B, right panel) aortic diameter and age and BSA**. Correlations are shown separately for TS with bicuspid (TS_bicuspid_) and tricuspid (TS_tricuspid_) aortic valves.

The aortic ratio was comparable in TS and controls, with the exception of a smaller ratio in TS at pos5 and pos6 (p= < 0.001) (Figure 2). At pos2 the mean ratio in TS was considered abnormal (1.52 ± 0.26), even though the ratio at this position was not significantly different from the ratio in the control population (p = 0.4). Ratios greater than >1.5 were found at one or more positions in 46/95 (48%) of TS women (pos1 (32%); pos2 (43%); pos3 (21%); pos4 (12%)). Nine out of 24 (37.5%) controls had an enlarged ratio at one or more positions.

### Aortic dilation

**Figure 3:**
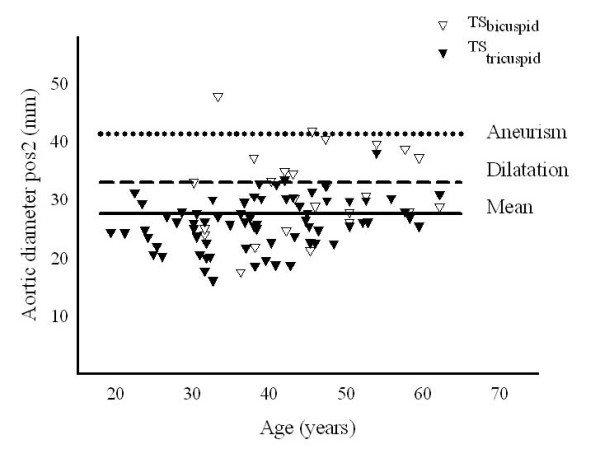
**Individual ascending aortic diameters (pos2) in TS by CMR**. The individuals are marked according to their aortic valve status.

On CMR, aortic dilation was found at one (or more) of 8 positions in 22 (23%) women with TS; amongst these, thirteen were dilated at two or more sites and 11 (85%) had BAV. Aortic diameter and enlargement at pos2 is depicted in figure 3. Three women with TS and BAV fulfilled the criteria for aortic aneurysm (Table 4), where dilation was present in 6/8 positions in one patient and in 4/8 positions in the other two women. Two control women (8%) presented with aortic dilation at one or more regions.

**Table 4:** TS with dilatation or aneurysm at the measuring positions.

	Dilatation	Aneurysm	χ^2^-test dilatation (BAV vs TAV)
**ECHOCARDIOGRAPHY **(n = 97)			
Aortic valve sinus	8 (6)	0	0.001
			
**CMR **(n = 94)			
Aortic valve sinus	11 (7)	2 (2)	0.003
Ascending aorta	12 (10)	0	< 0.001
Aorta proximal to innominate artery	11 (8)	0	< 0.002
Proximal transverse aortic arch	6 (5)	0	0.002
Distal transverse aortic arch	1 (1)	0	0.1
Aortic isthmus	1 (1)	0	0.1
Descending aorta	4 (0)	1 (1)	0.001
Descending aorta (diaphragm level)	3 (3)	0	0.004

Not all participants have obtained measurements at all positions. The first number depicts the total number with dilatation/aneurysm, while bracketed number represents those with dilatation or aneurysm and bicuspid aortic valve. The number of individuals with dilatation at only one position was 22 (23%) and at 2 positions or more was 13 (14%).

On echocardiography, dilation at the aortic sinus was found in 8 TS women (Table 4) and in two controls. All 8 patients also fulfilled the criteria for dilation in the thoracic aorta beyond the sinus when measured by CMR, whereas only 7/20 (35%) of the women with TS who had dilation at more than 1 position by CMR were identified by echocardiography. Both cases of aneurysm found on CMR were identified by echocardiography, and the control woman identified with dilation on CMR was also identified to have dilation on echocardiography.

### Variables predicting aortic diameter

In bivariate correlation analyses aortic diameter correlated to age at all positions in TS (R = 0.22-0.5, p < 0.03), but not to height, weight, or BMI. Correlation to BSA was only found at pos8 (R = 0.23; p = 0.02) but not at any other point of measurement. Correlation to BSA was found at pos7 (R = 0.21; p = 0.04) and pos8 (R = 0.23; p = 0.02). Karyotype correlated to aortic dimensions at pos1 (R = 0.28, p = 0.007): women with 45,X had a larger diameter at this position when compared with non-45,X (p = 0.007). An association to both systolic 24-h AMBP (pos3: R = 0.32, p = 0.002; pos5: R = 0.39, p < 0.001; pos6: R = 0.32, p < 0.001; pos8: R = 0.27, p = 0.01) and diastolic 24-h AMBP (pos3: R = 0.29, p = 0.006; pos5: R = 0.26, p = 0.015) was present at some positions. Pulse pressure did not correlate with aortic diameter. Earlier growth hormone, duration of estrogen deficit as well as current antihypertensive treatment correlated significantly to the diameter at several positions, albeit not when adjusting for age (data not shown).

Descending aortic diameters were increased in those with a coarctation repair when compared to those without such history (pos5 (p = 0.004); pos7 (p = 0.004)). No difference was seen in aortic diameters, when comparing TS women with webbed neck or low hairline to TS without such phenotypic traits.

### Multiple regression models

We constructed multiple linear regression models to incorporate age, BSA, systolic AMBP, diastolic AMBP, aortic valve morphology, and karyotype as independent variables and aortic diameter (CMR) as the dependent variable. Age, BSA, blood pressure, and aortic valve morphology emerged as independent explanatory variables at several positions. Karyotype was only an explanatory variable to pos1 (Table 5). The influence of these variables varied considerably with the position of measurement through the thoracic aorta. At position 6-7, the incorporated variables contributed with more than 50% of the variation in aortic diameter, while the explanatory variables contributed with 30-40% to the variation in CMR diameter at pos2-5. At pos1 only 11% of the variation in aortic diameter could be contributed to the explanatory variables identified in this study (Table 5).

**Table 5:** Multiple linear regression model including the independent variables: age, BSA, AMBP systolic (AMBPsys), AMBP diastolic (AMBPdia), bi- or tricuspid aortic valve (bi/tri), HRT deficiency, and karyotype.

Position CMR	Model		Significant variables	
	r	p		p
Aortic valve sinus (pos1)	0.33	0.026	BAV/TAV	0.026
Ascending aorta (pos2)	0.56	< 0.001	Age	0.006
			BAV/TAV	0.001
			AMBPdia	0.036
			BSA	0.019
Aorta proximal to innominate artery (pos3)	0.57	< 0.001	Age	0.001
			BAV/TAV	0.045
			AMBPdia	0.012
			BSA	0.002
Proximal transverse aortic arch (pos4)	0.66	< 0.001	Age	< 0.001
			BAV/TAV	0.004
			AMBPdia	0.053
			BSA	0.015
Distal transverse aortic arch (pos5)	0.64	< 0.001	Age	< 0.001
			AMBPdia	0.034
			BSA	< 0.001
Aortic isthmus (pos6)	0.75	< 0.001	Age	< 0.001
			BSA	< 0.001
Descending aorta (pos7)	0.66	< 0.001	Age	0.001
			BAV/TAV	< 0.001
			AMBPsys	0.032
			AMBPdia	0.004
			BSA	0.001
Descending aorta (pos8) (diaphragm level)	0.75	< 0.001	Age	< 0.001
			BAV/TAV	< 0.001
			AMBPdia	0.041
			AMBPsys	0.001
			BSA	< 0.001

## Discussion

The present study provides several cornerstone findings in TS: i) aortic valve configuration is a key determinant to aortic morphology, and BAV associates with the larger aortic diameter even beyond the ascending aorta; ii) aortic dilation can be present despite an average diameter comparable to that of healthy female controls; iii) aortic dilation is present in a substantial fraction of non-selected adult women with TS.

Recent observations unanimously confirm that women with TS are at increased risk of aortic dissection [1]; if this is universal to TS, or particularly relevant only to certain subgroups is not entirely resolved [1,16]. As in other populations facing an increased risk of aortic dissection [17,18], the presence of BAV may be a key risk factor in TS [1,16]. In daily clinical practice, prediction of the risk of dissection is based on the finding of a severely enlarged aorta or serial studies of aortic dimensions, where larger aortic caliber or high aortic growth rates predict increased risk. Here, the association is confirmed between BAV and a larger ascending aorta, which again would confer with an increased risk of dissection. Interestingly, in this population where up to a third of thoracic aortic dissections occur in the descending aorta [16], this association is extended into the transverse aortic arch and the descending aorta, and a further link is indicated between BAV and aortic coarctation in TS. It does, however, remain to be resolved if aortic dilation as encountered in TS can be attributed primarily to the aortopathy complex seen in other, non-syndromic BAV patients or if it is more directly related to the syndrome and the combined burden of potential cardiovascular risk factors such as hypertension, tachycardia, estrogen deficiency, growth-hormone deficiency, and impaired glucose tolerance [19,20].

Other studies have indicated that the process leading to aortic wall disease and dissection in TS is multifactorial, as BAV was present in 27% of dissections in TS [16], which does not differ from the 31% with BAV seen in a mixed group of 111 non-TS patients undergoing operation for ascending aortic aneurysm [21]. The significance of the combined impact of the syndrome related traits, beyond BAV and aortic coarctation, is further supported by the fact that previous studies have shown the 45,X karyotype to confer an increased risk of dissection [10,16,22], and with 45,X the risk of an adverse metabolic and cardiovascular phenotype is considered generally increased [3,8]. It is here confirmed that not only the 45,X but also elevated blood pressure (diastolic pressure especially) carries increased risk of larger aortic diameters in adult TS, which is in line with high blood pressure being a risk factor for ascending aortic dilation both in the general population [23] and in women with TS [24].

No clear evidence exists to define the precise timing for medical or surgical intervention in this high-risk patient group, where the normal range for aortic measurements remains to be determined. Aortic dilation in TS is intuitively considered the principal surrogate marker for the risk of dissection, but dissections occur even with normal aortic echocardiograms within less than 15 month prior to the event [16]. This may reflect the sensitivity of echocardiography; the value of echocardiography as a screening tool in the TS population may be suboptimal secondary to the widespread abnormal chest dimensions, rendering the achievement of appropriate echocardiographic acoustic windows difficult. In this study, echocardiography was sensitive to severe aortic dilatation (aneurism) but less sensitive to less progressed dilatation as well as to dilatation at more positions; this is in line with previous findings [25]. Alternatively, this striking lack of association between pathologic aortic enlargement and aortic dissection may reflect the complexity of defining the pathologically enlarged aorta in TS, where follow-up studies after thorough cardiovascular work-up are limited and have failed to identify aortic growth or certain subgroups facing increased risk of progression in aortic caliber [26]. Interestingly, the definition of normality is a key question extending beyond TS, as a recent study challenges the currently used clinically risk stratification for aortic dissection: severe aortic dilation (to a degree where surgical intervention would normally be recommended, i.e. diameters > 5.5 cm) was only present in 41% of aortic dissection patients (all without chromosomal disease), even if a larger diameter was present in patients with BAV or Marfan syndrome [27].

Different strategies have been proposed to circumvent the issue of defining pathologic aortic dimensions in TS, whereof BSA indexing as the aortic size index is widely used. A strong, consistent correlation between BSA and aortic diameter did, however, not appear in the current cohort, contradicting previous studies of TS [7,8]. This shortage of correlation is not a lack of statistical power, as we have previously documented such correlation in a pediatric population using the same imaging technique in a cohort of more than half the size [28], and other studies showing good correlation have not been considerably higher powered [14]. Hypothetically, a prominent association between aortic diameter and BSA observed in children, adolescents, and younger adults becomes less prominent with age; the multiple co-morbidities of TS potentially impact adversely on aortic dimensions, causing BSA to lose its significance over time. Such syndrome-associated co-morbidities could besides age, be elevated blood pressure, diabetes, BAV, aortic coarctation, or karyotype. Collectively, these complex interactions may not only cause aortic dimensions in adult TS to spread over a wider range (as seen in this population), but they may also weaken the predictive capacity of BSA to aortic dimensions in the older TS patient: the traits of TS impacting through childhood and adulthood on aortic size and becomes of higher importance than BSA. Therefore, the observed dissociation of a correlation between BSA and aortic diameter in this non-selected adult TS population, recruited from a non-cardiology ward, may reflect a state of progressed aortic disease. The significance of using BSA indexing to define the TS women with large aortic size (i.e. the risk of aortic dissection) remains to be investigated in follow-up studies. But our study indicates that other factors could be of equal importance in terms of defining the abnormal aorta in the individual patient. It further raises the question of the correction for this variable, not uniformly correlating linearly with aortic size, may not be entirely unproblematic, as other important factors may be overlooked.

Alternatively to indexing for BSA, adjusting for aortic dimensions in regions normally unaffected by the aortic disease (the distal descending aorta), to obtain an aortic ratio has been proposed. This measure may be more robust to changes with age and body size [24] and therefore more appropriate in TS. In contrast to previous observations in women with TS [8], this ratio was not increased in our cohort compared to controls. It is our belief, that this ratio is inherently flawed in TS because the descending aorta was found to be abnormal in a considerable number of patients, and therefore the ratio will underestimate the extent of aortic dilatation. Furthermore, the ratio was above the limit for pathologic dimensions (aortic ratio > 1.5) in 38% in the healthy controls.

Compared with CMR, echocardiography is universally accessible, quick, cheap, safe and never contraindicated. But CMR not only allows imaging of the entire thoracic aorta but also offers an exquisite image quality in almost all subjects, as also seen in this study in which CMR could be performed in 95/102 (93%) of the recruited subjects. With the now documented increased prevalence of aortic dilatation along the entire thoracic aorta as well as the previously documented increased prevalence of coarctation as well as the elongated transverse aortic arch [3], accurate visualization of the complete thoracic aorta is particularly relevant in clinical follow-up in TS. To improve imaging and avoid the use of contrast agents, we used a specific CMR sequence, allowing for contrast-free and free-breathing imaging of the entire thoracic aorta with a minimal acquisition time. Besides obtaining measurements of high inter- and intraobserver variability, as seen in our Bland-Altman variability analysis, this technique allowed post-processing in any 3D plane and provided optimal conditions for measuring a true, accurate aortic caliber. It may therefore be of value in cardiovascular follow-up in this high risk cardiovascular population, where the pathology appears of multifactorial nature and where knowledge of adequate follow-up regimens for specific TS subgroups are limited in the face of a shortage of studies of the aortic disease and events over time and after through cardiovascular characterization.

## Conclusion

In conclusion, aortic dilation and aneurysm, predominantly affecting the ascending aorta, are widespread in TS. The presence of BAV identifies a TS subgroup with significantly larger aortic dimensions; blood pressure, karyotype, and age are additional major determinants of thoracic aortic diameter. Although echocardiography in most cases accurately and sufficiently identifies dilation of the proximal part of the aorta, CMR is not only a more sensitive technique for assessing the ascending aorta but in particular provides high-quality imaging of the remaining part of the thoracic aorta where significant dilatation, native narrowing or residual obstructions after previous repair of coarctation pose an additional aortic burden, increasing the risk of dissection. CMR is therefore a key tool not only in diagnosis of aortic dilatation, or even dissection, but is also a pivotal part of follow-up in order to sufficiently assess aortic caliber over time.

## Competing interests

The authors declare that they have no competing interests.

## Authors' contributions

Echocardiography was performed by NA and KM. CMR data was handled by EP, KM and EL. CG and JC conceived the study, with contributions to design and coordination made by: CG, JC, KS, EP, EL, KH and AHo. BH and CG performed the statistical analysis. The initial manuscript was drafted by BH and CG, with substantial contributions from KM, KS, NA, KH and AHa. All authors read and approved the final manuscript.
